# Tumor-localized catalases can fail to alter tumor growth and transcriptional profiles in subcutaneous syngeneic mouse tumor models

**DOI:** 10.1016/j.redox.2023.102766

**Published:** 2023-06-05

**Authors:** Allison Sheen, Yash Agarwal, Keith M. Cheah, Sarah C. Cowles, Jordan A. Stinson, Joseph R. Palmeri, Hadley D. Sikes, K. Dane Wittrup

**Affiliations:** a Koch Institute for Integrative Cancer Research, Massachusetts Institute of Technology, Cambridge, MA, USA; b Department of Biological Engineering, Massachusetts Institute of Technology, Cambridge, MA, USA; c Department of Chemical Engineering, Massachusetts Institute of Technology, Cambridge, MA, USA

**Keywords:** Catalase, Tumor-localized, Cancer, Transcriptional profile, RNAseq, Redox-directed therapies

## Abstract

Catalase is an antioxidant enzyme that catalyzes the rapid conversion of hydrogen peroxide to water and oxygen. Use of catalase as a cancer therapeutic has been proposed to reduce oxidative stress and hypoxia in the tumor microenvironment, both activities which are hypothesized to reduce tumor growth. Furthermore, exposing murine tumors to exogenous catalase was previously reported to have therapeutic benefit. We studied the therapeutic effect of tumor-localized catalases with the aim to further elucidate the mechanism of action. To do this, we engineered two approaches to maximize intratumoral catalase exposure: 1) an injected extracellular catalase with enhanced tumor retention, and 2) tumor cell lines that over-express intracellular catalase. Both approaches were characterized for functionality and tested for therapeutic efficacy and mechanism in 4T1 and CT26 murine syngeneic tumor models. The injected catalase was confirmed to have enzyme activity >30,000 U/mg and was retained at the injection site for more than one week *in vivo*. The engineered cell lines exhibited increased catalase activity and antioxidant capacity, with catalase over-expression that was maintained for at least one week after gene expression was induced *in vivo*. We did not observe a significant difference in tumor growth or survival between catalase-treated and untreated mice when either approach was used. Finally, bulk RNA sequencing of tumors was performed, comparing the gene expression of catalase-treated and untreated tumors. Gene expression analysis revealed very few differentially expressed genes as a result of exposure to catalase and notably, we did not observe changes consistent with an altered state of hypoxia or oxidative stress. In conclusion, we observe that sustained intratumoral catalase neither has therapeutic benefit nor triggers significant differential expression of genes associated with the anticipated therapeutic mechanism in the subcutaneous syngeneic tumor models used. Given the lack of effect observed, we propose that further development of catalase as a cancer therapeutic should take these findings into consideration.

## 1 Introduction

Catalase regulates reactive oxygen species (ROS) levels in aerobic organisms by catalyzing the conversion of hydrogen peroxide to water and oxygen [1]. Administration of exogenous catalase as a cancer therapeutic has been proposed to modulate the tumor microenvironment (TME) by: 1) decreasing oxidative stress by reducing the level of hydrogen peroxide and 2) decreasing hypoxia by increasing the amount of oxygen [[2], [3], [4], [5], [6], [7], [8], [9], [10]]. Both mechanisms are proposed to inhibit tumor growth.

Hypoxia is common in solid tumors and has long been an attractive target for cancer therapy [11,12]. It is known to aid in tumor progression and resistance to therapy by contributing to angiogenesis, altered metabolism, metastasis, evasion of immune responses, chemoresistance, and radioresistance [[12], [13], [14], [15], [16], [17]]. In the presence of tumor endogenous hydrogen peroxide, it has been proposed that catalase can generate oxygen, potentially decreasing hypoxia [[2], [3], [4], [5]].

Many cancer cell types exhibit elevated ROS levels and production of hydrogen peroxide due to metabolic and signaling abnormalities [18,19]. ROS production increases due to acquisition of oncogenic mutations, loss of tumor suppressors, metabolic hyperactivity, and hypoxic adaptations [20]. Additionally, some cancer cells have a decreased ability to metabolize hydrogen peroxide, leading to an excess of hydrogen peroxide relative to normal cells [21]. Hydrogen peroxide in particular is known to affect numerous intracellular signaling pathways [22], regulating processes such as cell proliferation, differentiation, migration, cell death, oxygen sensing, angiogenesis, tumorigenesis, and immune function [[22], [23], [24], [25]]. Hence, reduction of hydrogen peroxide by catalase could slow tumor growth by disrupting these pro-tumorigenic signaling pathways.

While ROS are known to promote pro-tumorigenic signaling, ROS can also promote anti-tumorigenic signaling and cause cancer cell death mediated by oxidative stress [20]. Based on this dual activity of ROS, catalase may play a dichotomous role in cancer, acting to suppress or promote tumor growth and metastasis depending on the metabolic landscape and redox status of cancer cells [26]. Studies have previously shown downregulation of catalase in some cancers and upregulation of catalase in other cancers is associated with tumor progression and metastasis. Furthermore, preclinical studies and clinical trials have shown that dietary antioxidant supplements can increase tumor growth, metastasis, or incidence of cancer [[27], [28], [29], [30]]. Catalase may exert differential effects on tumor growth and metastasis depending on the balance of pro- and anti-tumorigenic activities of hydrogen peroxide in cancer cells within the tumor.

Previous studies have shown that catalase can contribute to efficacy in certain mouse tumor models, but the results, context, and mechanistic explanations are inconsistent across studies [[2], [3], [4], [5], [6], [7], [8], [9], [10]]. Interpreting the function of catalase is often further complicated by the inclusion of other therapeutic agents. In metastatic models, catalase has been demonstrated to reduce the number of disseminated tumor cells and prolong survival, an action attributed to depletion of hydrogen peroxide [[8], [9], [10]]. In flank tumor models, catalase has not shown efficacy as a monotherapy, but produced cures in combination with radiation, which was attributed to hypoxia reduction [2,3]. A key component to many of these studies is attempting to maximize and maintain catalase exposure at the tumor site.

We studied the efficacy of catalase cancer therapy in subcutaneous syngeneic mouse tumor models with a further aim to elucidate potential mechanisms of action. We maximized intratumoral catalase exposure by injecting engineered catalase anchored to co-injected aluminum hydroxide (alum) particles; a strategy we previously showed enables weeks of intratumoral retention [31,32]. In addition to extracellular anchored catalase, we overexpressed intracellular catalase in tumor cell lines, with the aim of disrupting peroxide-mediated intracellular signaling [22].

In this work, we evaluate the efficacy of tumor-localized extracellular and intracellular catalase in subcutaneous syngeneic mouse tumor models. These models are non-metastasizing and therefore these studies do not address potential impacts of catalase on metastasis. Despite prolonged tumor-localized catalase activity, catalase alone or in combination with radiation did not improve treatment efficacy, as measured by primary tumor growth and survival, contrasting with prior studies [2,3]. Furthermore, increased catalase exposure failed to induce detectably altered gene expression programs attributable to either decreased oxidative stress or hypoxia.

## 2 Materials and methods

**Mice.** Female Balb/C (Taconic, BALB-F), C57BL/6 (Taconic, C57BL/6NTac), and albino B6 (JAX, 000058) mice were purchased and maintained in the animal facility at MIT. Six-to 12-week old mice were used in experiments. All animal studies and procedures were carried out following federal, state, and local guidelines under an institutional animal care and use committee-approved animal protocol by the Committee of Animal Care at MIT.

**Cell culture and stable-line generation.** 4T1 (American Type Culture Collection [ATCC]) and CT26 (ATCC) were cultured in Roswell Park Memorial Institute (RPMI) 1640 medium (ATCC) supplemented with 10% fetal bovine serum (FBS, Gibco). HEK293-FT (Invitrogen), B16F10 (ATCC), MC38 (a gift from J. Schlom, National Cancer Institute, Bethesda, MD), and B16F19 Trp2KO [33] were cultured in Dulbecco's modified Eagle's medium (DMEM, ATCC) supplemented with 10% FBS. All cells were cultured at 37 °C and 5% CO_2_. 4T1 and CT26 cell lines with doxycycline (DOX)-inducible expression of GFP (control) or CAT and GFP (CAT) were generated by lentiviral transduction. All cell lines tested negative for mycoplasma.

**Cloning and plasmid preparation.** A DNA sequence encoding human catalase (CAT) was purchased as a gBlock gene fragment (IDT). CAT was cloned with or without an C-terminal LPSTG motif for sortase-mediated transpeptidation and both were cloned with 6-His tag into the pE vector (sequences in SI) using In-Fusion cloning (Takara Bio). Sortase A pentamutant (eSrtA) in pET29 was a gift from David Liu (Addgene plasmid #75144). Plasmids were then transformed and amplified in Stellar cells for sequencing or in Rosetta 2 DE3 *E. coli* cells (Novagen) for protein expression. GFP or CAT and GFP (separated by a T2A peptide) (sequences in SI) were cloned into the pCW57.1 lentiviral vector, a doxycycline (DOX) inducible vector for mammalian expression. pCW57.1 was a gift from David Root (Addgene plasmid #41393). Plasmids were transformed and amplified in One Shot Stbl3 cells (Invitrogen).

**Protein expression and purification**. Rosetta cells were transformed, grown to an optical density (OD_600_) of 0.4–0.6, induced with IPTG (0.5 mM), and transferred to 30 °C overnight. Cell pellets were isolated via centrifugation, resuspended in sonication buffer (3% glycerol, 1% Triton-X-100, 300 mM NaCl, 50 mM NaPO_4_, pH = 8) with protease inhibitor (Roche), and sonicated at 4 °C. Lysate was centrifuged at 15,000×*g* for 30 min.

Protein was purified from the filtered supernatant by gravity column using Ni-NTA resin (HisPur™ Thermo Scientific) followed by size-exclusion chromatography using a Superdex® 200 Increase 10/300 GL (Cytiva) column on an AKTA fast protein liquid chromatography system (GE Healthcare). To remove endotoxin, proteins were immobilized on Ni-NTA resin and washed with 0.1% Triton-X-114 in PBS. Proteins were buffer exchanged into tris-buffered saline (TBS) or phosphate-buffered saline (PBS) using Amicon filters (EMD Millipore). Proteins were 0.2 μm-filtered and confirmed for minimal endotoxin (Endosafe®-PTS endotoxin test, Charles River Laboratories). Molecular weight was confirmed by running proteins alongside a Novex Sharp Pre-Stained Protein Standard on a NuPAGE 4–12% Bis-Tris gel (Invitrogen) with MES buffer and SimplyBlue Safe Stain (Life Technologies). Proteins were flash frozen in liquid nitrogen and stored at −80 °C.

**Cell viability H**_**2**_**O**_**2**_**challenge**. Cells were plated at 10,000 cells/well in a 96-well plate, then 24h later, media was supplemented with 0 or 2000 ng/mL doxycycline (DOX, Sigma-Aldrich). 24h later, media was replaced with media +1 mM H_2_O_2_. After 1h exposure to H_2_O_2_, cell viability was measured using CellTiter-Glo® 2 (Promega). Luminescence was quantified using a Tecan Infinite M200 Pro plate reader.

**Catalase activity assay.** Catalase activity was measured using the Góth method [34]. Briefly, 20 μL catalase-containing sample was mixed with 100 μL 65 mM hydrogen peroxide in PBS, incubated for 1 min, then stopped by addition of 100 μL 32.4 mM ammonium molybdate in PBS. Hydrogen peroxide complexes with ammonium molybdate producing an absorbance at 405 nm, which was quantified using a Tecan Infinite M200 Pro plate reader. A405 was converted to H_2_O_2_ concentration using a standard curve. Catalase activity is quantified as mol H_2_O_2_ consumed per min per mole catalase, or mol H_2_O_2_ consumed per min if catalase quantity is unknown. Catalase purified from bovine liver (Sigma-Aldrich, C40) was used as a control.

**Western blot.** Cells (*in vitro*) were lysed with RIPA buffer (Abcam), centrifuged, and supernatant was filtered. Tumor samples to be analyzed were homogenized via physical disruption in T-PER buffer (Thermo Scientific) and protease inhibitor. The tumor lysate was analyzed as is (for extracellular, alum-bound protein analysis) or centrifuged and the supernatant was filtered and collected (for intracellular protein analysis). Protein concentrations were determined using Pierce BCA kit (Thermo Scientific). 1–2 μg of purified protein or 20–50 μg cell lysate was resolved by electrophoresis (4–12% Bis-Tris gel, Invitrogen), transferred to nitrocellulose, and probed with anti-catalase (1:500, Biolegend-863301), anti-beta-actin (1:500, Biolegend 643801), and/or anti-phosphotyrosine (1:1000, Abcam 179530) antibodies. Proteins were visualized using IRDye800CW goat anti-mouse or IRDye680RD goat anti-rabbit IgG secondary and Odyssey Imager (LI-COR).

**In vitro alum binding.** Alum used was Alhydrogel (Invivogen). Proteins were conjugated to Alexa Fluor 647 (AF647) via NHS labeling (Invitrogen). Free dye was removed via buffer exchange using Amicon® Centrifugal Filters. Fluorescently labeled CAT proteins (20 μg) and alum (100 μg) were incubated for 20 min in TBS and rotated at room temperature. The sample was centrifuged for 10 min at 15,000×*g* to pellet alum, the supernatant was aliquoted, and the pellet was resuspended with PBS +10% mouse serum. The tubes were rotated at room temperature. At the indicated time points, samples were centrifuged, and supernatant was aliquoted and replaced with PBS +10% mouse serum. Supernatant fluorescence was measured using a plate reader and converted to alum-bound protein (%) using a standard curve of AF647-labeled protein vs. fluorescence.

**IVIS imaging study.** Proteins were fluorescently labeled as described above. On day 0, albino C57BL/6 mice on alfalfa-free feed were inoculated with 1 × 10^6^ apigmented B16F10 Trp2KO cells s.c. in the right flank. After the tumors were palpable (day 6), tumors were treated intratumorally (i.t.) and non-tumor bearing mice were treated s.c. with 20 μL of AF647-labeled CAT proteins (20 μg) with or without alum (100 μg). Fluorescence at the injected site was imaged using an *in vivo* imaging system (IVIS, Perkin-Elmer) with 640-nm excitation and 680-nm emission filters. Image analysis was done using Living Image (Caliper Life Sciences), and data were normalized to the maximum signal throughout the experiment per protein.

**Tumor treatments and survival.** On day 0, mice were inoculated with 1 × 10^6^ CT26, CT26-CAT, CT26-control, MC38, or B16F10 cells or 0.5 × 10^6^ 4T1, 4T1-CAT, or 4T1-control cells in 50 μL PBS subcutaneously (s.c.) in the right flank. Treatments began on day 5 when tumors were palpable. Mice were sorted into groups to ensure similar starting average tumor area. The dose of CAT-ABP given was 20 μg, similar to the previously reported dosing [2]. The dose of alum given was 100 μg [31]. CAT-ABP and alum were mixed and delivered in TBS. CAT-ABP and alum were dosed every 7 days until euthanasia or a maximum of 4 total doses. For intracellular catalase cell line studies, mice were fed a DOX diet (625 mg/kg, Envigo Teklad) starting on day 5 for a maximum of 4 weeks or until euthanasia. For radiation combination experiments, tumors were treated first with i.t. CAT-ABP and alum or alum alone, followed by radiation. Local tumor radiation (6 or 9 Gy dose) was delivered using an x-ray radiation source (XRad320, Precision X-Ray) and a 1 cm diameter exposure field centered on the tumor. For all tumor studies, tumor area (length x width) and body weights were monitored. Mice were euthanized when tumors exceeded 100 mm^2^.

**RNA purification, sequencing, and analysis.** Whole tumor tissue was homogenized and lysed using gentleMACS M tubes (Miltenyi Biotec) and the RNA_01 program. After homogenization/lysis, samples were run through QiaShredder and RNeasy kit (Qiagen) to purify RNA according to manufacturer's instructions. RNA quality was assessed using a Fragment Analyzer (Agilent). Library prep was performed using the High Throughput 3’ Digital Gene Expression (HT3DGE) method. Sequencing was performed on a Illumina NextSeq system. Raw FASTQ sequencing data was converted to reads per gene using the Smart-seq2 pipeline within TerraBio (Broad Institute). Aligned read data was analyzed in R using DESeq2 [35]. Bulk RNA sequencing was performed with *n* = 5–6 biological replicates/samples per group and 2 technical replicates per sample. After quality control analysis, some technical replicates were excluded due to low read depth.

**Statistics.** Statistics were performed using Prism (GraphPad). Survival was compared by log-rank Mantel-Cox test. As described in figure captions, other metrics were compared by one-way analysis of variance (ANOVA) with Tukey's multiple comparisons test. The *n* and *P* values are indicated in figure legends or captions.

**Data availability.** Bulk RNA sequencing data is publicly available in Gene Expression Omnibus (GEO) at GSE225211.GEO. Additional data that support the findings of this study are available from the corresponding author, KDW, upon reasonable request.

## 3 Results

### 3.1 Development and characterization of tumor-localized extracellular catalase

**Fig. 1:**
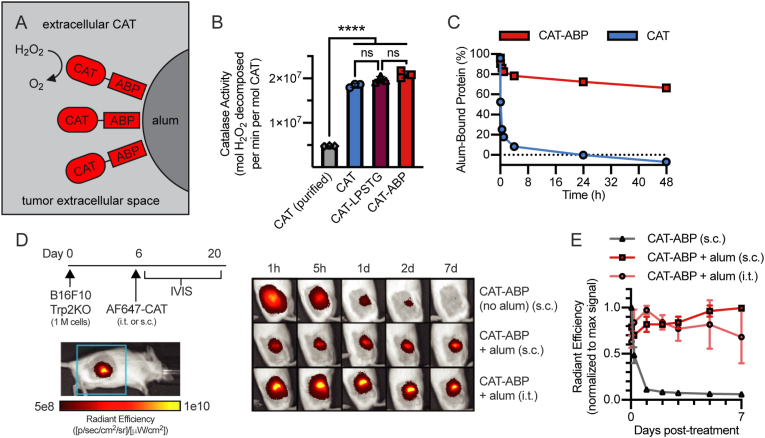
**Characterization of activity and tumor-localization of extracellular catalase. (A)** Approach for extracellular, tumor-localized catalase (CAT). Recombinant CAT fused to an alum-binding peptide (ABP) enables binding to the surface of alum, an injectable particulate material. **(B)** Catalase activity of recombinant catalase proteins compared to a commercial CAT purified from bovine liver. Catalase activity was quantified using the Góth method [34]. **(C)** AF647-labeled CATs and alum were incubated for 30 min in TBS, then resuspended in PBS + 10% mouse serum at t = 0 and replaced at the indicated time points. Fluorescence spectroscopy was used to measure the percentage of protein bound to alum over time; mean±SD (*n* = 3). **(D**–**E)** Non-tumor bearing mice were injected subcutaneously (s.c.) or mice bearing subcutaneous B16F10 Trp2KO tumors were injected intratumorally (i.t.) on day 6 with 20 μg AF647-labeled CAT-ABPs ± 100 μg alum and tracked by IVIS. **(D)** Representative images. The blue box shows the field of view around the tumor. **(E)** Total radiant efficiency at the injection site normalized to max signal; mean±SD (*n* = 3–4). h, hours; d, days. Statistics: catalase activity compared by one-way ANOVA with Tukey's multiple comparisons test. ns, not significant; *P < 0.05; **P < 0.01; ***P < 0.001; ****P < 0.0001. (For interpretation of the references to color in this figure legend, the reader is referred to the Web version of this article.)

Extracellular catalase (CAT) was anchored to alum particles via an alum-binding peptide (ABP), which is composed of phosphorylated residues that undergo a ligand exchange reaction with the surface hydroxyls on alum (Fig. 1A) [36,37]. Based on prior work [31,32], we hypothesized that intratumoral injection of alum-anchored CAT-ABP would enable >1 week of retention. To generate CAT-ABP, catalase was recombinantly expressed and fused to ABP using a sortase-mediated transpeptidation reaction (sortagging) (SI Fig. 1A–C). Recombinant fusion of an C-terminal LPSTG motif to CAT enables recognition by the sortase enzyme, which can then attach an ABP (pTyr_4_) modified with an N-terminal poly-glycine motif [[38], [39], [40]]. The sortagged product (CAT-ABP) was purified from the reaction mixture and presence of the phosphorylated peptide in the purified product was confirmed by anti-pTyr western blot (SI Fig. 1D and E). Activity of the recombinant CATs was measured and compared to a purified, commercial CAT (Fig. 1B). CAT, CAT-LPSTG, and CAT-ABP exhibit similar enzyme activity to one another, all with 3–4 fold higher activity than purified, commercial CAT (≥10,000 U/mg), confirming that addition of the LPSTG motif and subsequent attachment of the ABP to CAT does not negatively impact enzyme activity. To evaluate the alum-binding functionality of CAT-ABP, an *in vitro* alum-release assay was performed comparing the ability of CAT-ABP and unmodified CAT to bind alum in serum-containing PBS over time. While CAT and CAT-ABP exhibit similar initial adsorption to alum, CAT rapidly desorbs from alum in serum-containing PBS, while the majority of CAT-ABP protein remains bound to alum over time.

Next we evaluated the *in vivo* retention of CAT-ABP. Alexa Fluor 647 (AF647)-labeled CAT-ABP was injected subcutaneously (s.c) in non-tumor bearing mice or i.t. into apigmented B16F10 tumors and the retention at the injection site was tracked for 7 days (Fig. 1D and E). When injected with alum, CAT-ABP was retained at the injection site for all 7 days. In contrast, when injected without alum, CAT-ABP is almost entirely absent from the injection site within 1 day. We confirmed that the retained protein is functional by measuring catalase activity of homogenized tumor samples 4 days after i.t. injection (SI Fig. 2A).

### 3.2 Development and characterization of tumor-localized intracellular catalase

**Fig. 2:**
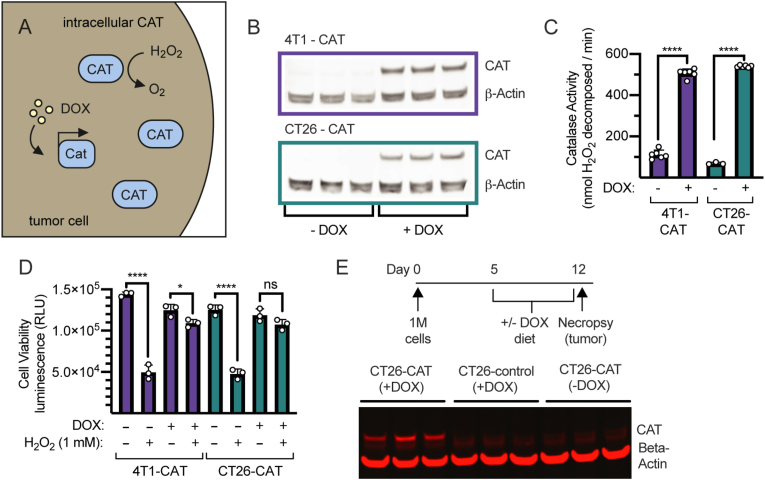
**Characterization of expression and activity of intracellular catalase. (A)** Approach for intracellular, tumor-localized CAT. Tumor cell lines were engineered for doxycycline (DOX)-inducible intracellular CAT overexpression. **(B)** Western blot of catalase and beta-actin (loading control) in cell lysate of 4T1-and CT26-CAT cells cultured ± 2000 ng/mL DOX for 24 h; (*n* = 3 shown) **(C)** Catalase activity in cell lysate. **(D)** Cell viability after exposure to high-concentration hydrogen peroxide. 4T1-and CT26-CAT cells were cultured ± 2000 ng/mL DOX for 24 h, exposed to 1 mM H_2_O_2_ in media for 1 h, then cell viability was measured using CellTiter-GLO luminescence cell viability assay. **(E)** Mice inoculated with CT26-CAT or CT26-control tumor cell lines were placed on a 625 mg/kg DOX diet (+DOX) on day 5 or kept on a regular diet (-DOX), maintained on those diets for 7 days, then tumors were harvested for analysis. Catalase expression was measured in homogenized tumor lysate by western blot (*n* = 3 biological replicates shown). Statistics: catalase activity and cell viability compared by one-way ANOVA with Tukey's multiple comparisons test. ns, not significant; *P < 0.05; **P < 0.01; ***P < 0.001; ****P < 0.0001.

We also engineered the 4T1 and CT26 tumor cell lines to over-express catalase in a doxycycline (DOX)-inducible manner (Fig. 2A), by lentiviral transduction. Catalase expression was localized to the cytoplasm by removal of the native peroxisomal localization motif (KANL) at its C terminus [41,42]. 4T1 and CT26 cell lines were chosen because previous studies have shown that catalase can contribute to slower tumor growth in combination with radiation and/or chemotherapy in these tumor models [[2], [3], [4]]. Control cell lines that only express intracellular GFP were generated in parallel. Both 4T1-CAT and CT26-CAT cell lines show DOX-dependent CAT expression by western blot (Fig. 2B) and accompanying increases in catalase activity (Fig. 2C). To evaluate the functional differences that result from increased intracellular CAT-expression, we measured cell viability after exposure to high concentration (1 mM) hydrogen peroxide (H_2_O_2_) (Fig. 2D). While cells with only endogenous CAT expression (-DOX) show a 60–70% decrease in cell viability after 1 h exposure to H_2_O_2_, cells with increased CAT expression (+DOX) have significantly enhanced antioxidant capacity, showing only a 9–13% decrease in viability.

To characterize CAT expression of these cells *in vivo*, mice were inoculated with CT26-CAT or CT26-control cells, then placed on a doxycycline-diet (+DOX) 5 days later or maintained on a regular diet (-DOX). After 1 week, CAT expression in homogenized tumor lysate was measured by western blot (Fig. 2E). Increased CAT expression was observed specifically in CT26-CAT (+DOX) lysate compared to CT26-CAT (-DOX) and CT26-control (+DOX) lysates, confirming DOX-induced CAT expression for at least a week *in vivo*. Finally, we measured the catalase activity of these lysates and observed an increase in catalase activity in the CT26-CAT (+DOX) lysate compared to CT26-CAT (-DOX) and CT26-control (+DOX) lysates, confirming that DOX-induced CAT expressed is enzymatically active *in vivo* (SI Fig. 2B).

### 3.3 Intratumoral catalase therapies fail to modify tumor growth

**Fig. 3:**
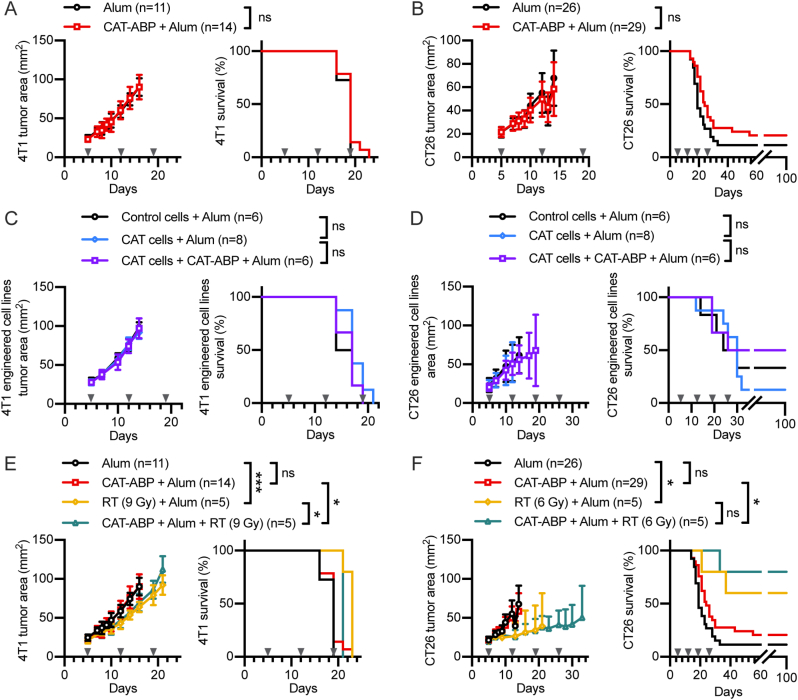
***In vivo* efficacy of tumor-localized catalase in murine tumor models**. **(A**–**F)** Mice were inoculated on day 0 with 0.5 × 10^6^ 4T1 or 1 × 10^6^ CT26 cells. Mice were treated i.t. on days 5, 12, 19, 26 (triangles above x-axis) with 20 μg CAT-ABP and 100 μg alum or alum alone in 20 μL TBS. **(A**–**B)** Tumor growth and survival for wildtype (WT) **(A)** 4T1 and **(B)** CT26 treated with CAT-ABP and alum or alum only. **(C**–**D)** Tumor growth and survival for **(C)** 4T1-and **(D)** CT26-CAT and -control tumor models. Mice were fed a doxycycline diet starting on day 5 for up to 4 weeks to turn on CAT expression and treated with CAT-ABP and alum or alum only. **(E**–**F)** Tumor growth and survival for WT **(E)** 4T1 and **(F)** CT26 tumors treated with CAT-ABP and alum or alum only, combined with radiation therapy (RT). RT was performed on day 5, following initial i.t. injection. Statistics shown are for survival. Statistics: survival compared by log-rank Mantel-Cox test. ns, not significant; *P < 0.05; **P < 0.01; ***P < 0.001.

Both catalase therapies, extracellular and intracellular, were first evaluated for therapeutic efficacy as single agents. Mice bearing established 4T1 breast tumors and CT26 colorectal carcinoma tumors were treated with 20 μg CAT-ABP + alum i.t. weekly starting on day 5 for 4 doses or until euthanasia. Tumor growth and survival (Fig. 3A and B) were not significantly different between treated and untreated mice, in either tumor model (*P* = 0.3465 for 4T1 and *P* = 0.1084 for CT26 survival). We also treated mice bearing MC38 colon carcinoma tumors and B16F10 melanoma tumors and observed no efficacy (*P* = 0.3924 for MC38 and *P* = 0.3014 for B16F10 survival) (SI Fig. 3).

Next, we inoculated mice with the tumor cell lines expressing intracellular catalase (4T1-or CT26-CAT) or the control (4T1-or CT26-control). After 5 days, the mice were placed on a doxycycline-diet (+DOX) and maintained on the diet for up to 4 weeks. We also tested a combination of both extracellular and intracellular catalase, treating tumors expressing CAT with CAT-ABP + alum as before. Tumor growth and survival (Fig. 3C and D) demonstrated that neither the expression of intracellular catalase nor a combination of intra- and extracellular catalase had observable efficacy in either tumor model.

After observing no catalase monotherapy efficacy, we tested extracellular catalase in combination with radiation therapy (RT), a combination previously reported to be efficacious in CT26 and 4T1 tumor models [2,3]. Mice bearing established 4T1 or CT26 tumors were treated with CAT-ABP + alum as before and given a single dose of x-ray RT (6–9 Gy) localized to the tumor on day 5 following the first dose of catalase. While RT improved survival (Fig. 3 E-F) in both models (*P* = 0.0005 for 4T1 and *P* = 0.0201 for CT26), the combination of CAT-ABP + alum and RT did not slow tumor growth or prolong survival compared to RT alone in either model.

### 3.4 Tumor-localized catalase therapies fail to modify tumor transcriptional profiles

**Fig. 4:**
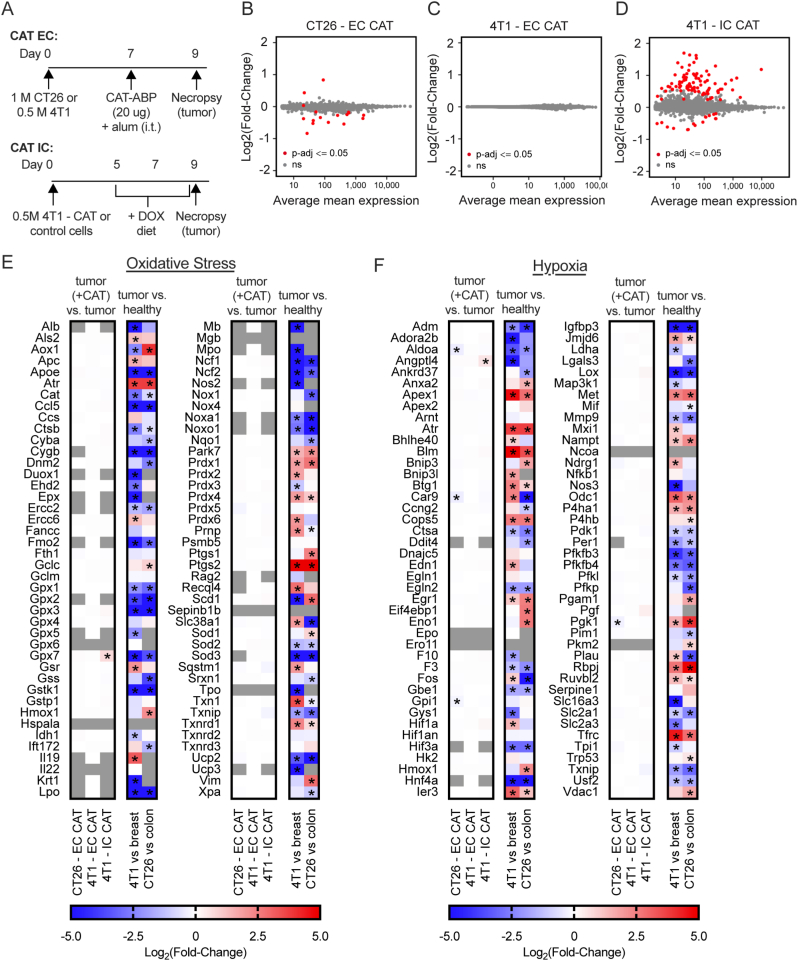
**RNA sequencing of murine tumors treated with tumor-localized catalase.** For treatment with extracellular catalase (EC CAT), mice were inoculated on day 0 with 1 M CT26 or 0.5 M 4T1 cells and treated i.t. on day 7 with CAT-ABP + alum or alum only. For treatment with intracellular catalase (IC CAT), mice were inoculated on day 0 with 4T1-CAT or 4T1-control cells and started on a DOX diet on day 5, then treated i.t. on day 7 with alum. On day 9 for both EC and IC CAT, tumors were excised and RNA was extracted for bulk RNA sequencing. *n* = 5–6 per group. (**A**) Study timelines. (**B-D**) Differential gene expression shown as MA plots with fold-change (y-axis) plotted against mean normalized count/expression (x-axis), where each dot represents a single gene and significant (*p*-adj ≤0.05) differentially expressed genes are shown in red. MA plots of (**B**) extracellular catalase (CAT-ABP + alum) in CT26 relative to alum-only control (CT26 – EC CAT), (**C**) extracellular catalase (CAT-ABP + alum) in 4T1 relative to alum-only control (4T1 – EC CAT), and (**D**) intracellular catalase in 4T1-CAT relative to 4T1-control (4T1 – IC CAT). (**E-F**) Fold-change of genes associated with (**E**) oxidative stress [45] and (**F**) hypoxia [44] from differential gene expression analysis comparing CAT-treated tumors and untreated tumor controls (left) and tumor cells and healthy tissue data from literature (right) [[46], [47]]. Healthy tissue comparisons for 4T1 and CT26 cells were mammary gland (breast) and colon respectively. Significant (*p*-adj ≤0.05) differentially expressed genes are noted with an asterisk. (For interpretation of the references to color in this figure legend, the reader is referred to the Web version of this article.)

Although our catalase interventions were not efficacious in the tumor models we tested, we sought evidence for altered gene expression resulting from them by bulk RNA sequencing as an unbiased approach to evaluate what, if any, changes are associated with increasing tumor-localized extracellular or intracellular catalase activity. To evaluate extracellular catalase (EC CAT), mice bearing established 4T1 breast tumors and CT26 colorectal carcinoma tumors were treated with 20 μg CAT-ABP + alum or alum alone i.t. on day 7, then 2 days later, tumors were harvested and RNA was extracted for sequencing (Fig. 4A, top). To evaluate intracellular catalase (IC CAT), mice bearing established 4T1-CAT or 4T1-control tumors were placed on a DOX-diet on day 5, given 2 days for systemic DOX concentrations to increase and equilibrate [43], then after another 2 days, tumors were harvested and RNA was extracted for sequencing (Fig. 4A, bottom). Differential gene expression analysis was performed on the RNA sequencing data using *DESeq2* [35]. Tumors treated with catalase were compared to their appropriate non-CAT treated control. The fold-change and mean normalized expression were plotted for each gene, with the significant (*p*-adj ≤0.05) genes highlighted in red (Fig. 4B–D).

Treatment with extracellular catalase in CT26 induced minimal changes, with only 17 genes with statistically significant (albeit quantitatively small) differential expression (Fig. 4B, SI Table 2). Three of the significant genes, *Aldoa*, *Pgk1*, and *Car9*, are known to be responsive to hypoxia [44]. Their expression decreases 15–30% in response to EC CAT, suggesting catalase may result in very subtle increases in tumor oxygen levels. None of the other significantly differentially expressed genes were associated with known catalase activity. Extracellular catalase in 4T1 (4T1 – EC CAT) did not result in any genes with significant differential expression (Fig. 4C). Increased intracellular catalase in 4T1 (4T1 – IC CAT) resulted in 105 genes with significant differential expression (Fig. 4D, SI Table 2). Within these genes, we did not find any sets of genes related to direct or indirect effects of catalase.

To determine if there were more subtle trends specifically in genes associated with oxidative stress or hypoxia, we looked at the fold-change values for genes associated with these phenotypes across groups (Fig. 4E and F) [44,45]. Despite a few significantly differentially expressed genes, we do not observe a larger signature across these gene sets that would suggest catalase is significantly modifying the level of hydrogen peroxide or oxygen in these tumor models. To put these minimal changes into context, we analyzed gene expression data from the literature comparing CT26 and 4T1 tumor cells in culture to their corresponding normal mouse tissue, colon and mammary gland/breast for CT26 and 4T1 respectively, and plotted the fold-change values in the right two columns of Fig. 4E and F [46,47]. Our data suggest these tumor models exhibit gene expression programs consistent with oxidative stress and hypoxia – however, our interventions significantly increasing extracellular and/or intracellular catalase made no detectable difference to these responses.

## 4 Discussion

Previous studies have suggested that exogenous catalase can contribute to efficacy in mouse tumor models [[2], [3], [4], [5], [6], [7], [8], [9], [10]]. The therapeutic action of catalase has been attributed to direct consequences of its enzymatic activity, either through increasing oxygen or decreasing hydrogen peroxide in the tumor microenvironment. Contrary to our expectations from prior literature, we did not observe any statistically significant effect from tumor-localized catalases. Here, we report a comparison and analysis of *in vivo* efficacy and gene expression for tumor-localized catalases in two different compartments, either injected and retained in the tumor (extracellular) or over-expressed by tumor cells (intracellular) (Figs. 1 and 2). Despite observing long-lived catalase presence and activity at the tumor, neither extracellular or intracellular catalases altered tumor growth or gene expression associated with hypoxia or oxidative stress (Figs. 3 and 4).

While single-agent catalase was not necessarily expected to slow growth in flank tumors, it was surprising that the addition of extracellular catalase to radiation therapy did not result in additional tumor delay (Fig. 3E and F), as this combination has previously shown efficacy in the same tumor models [2,3]. Although the therapeutic components used herein are not identical to those used previously, the dose and activity of catalase and radiation dosing are similar. A 20 μg dose of catalase with activity >35,000 U/mg was used previously, compared to our 20 μg dose with activity 3–4 fold higher than a commercial CAT with activity >10,000 U/mg (Fig. 1B) [2]. The radiation dosing used in a previous study was 6 Gy, compared to a 6–9 Gy dose used here [3]. One difference of our approach for tumor-localized extracellular catalase is the use of alum-anchoring. While anchoring to alum robustly enhances retention of catalase activity in the tumor (Fig. 1D and E), it may result in an uneven distribution. Immediately after injection, alum is known to distribute throughout the tumor, but 3 days later the majority of the alum is found aggregated into a large depot [31,48]. This behavior may result in high catalase activity near the alum depot and low activity in other areas. The majority of alum-bound catalase is expected to remain extracellular, but we cannot exclude the possibility that some alum and alum-bound protein may be internalized, as this has been previously reported in antigen presenting immune cells [37,48]. Previous data from our lab in the form of tumor histology, performed on tumors 7 days after treatment with alum-bound protein, suggests the vast majority of protein is outside the cells [48].

Although the intracellular catalase approach does not achieve the same level of additional catalase activity in the tumor as with extracellular catalase, it does elevate catalase activity throughout the tumor. Furthermore, it provides increased catalase activity in the cytosol to interdict and scavenge hydrogen peroxide at the site of hydrogen peroxide-mediated cell signaling. While the catalase expression level achieved by these tumor cells is sufficient to significantly protect cells from an insult of high exogenous hydrogen peroxide *in vitro* (Fig. 2D), the increase in intracellular scavenging of hydrogen peroxide in tumor cells *in vivo* neither alters tumor growth nor oxidative stress response or hypoxia gene expression programs. Our results may be influenced by the cytosolic localization of catalase. Future work localizing catalase to other subcellular compartments such as mitochondria, the nucleus, or tethering to the plasma membrane may produce different effects and further elucidate the spatial importance of antioxidant expression and hydrogen peroxide localization in tumor growth. The utilization of doxycycline (DOX) to stimulate gene expression may also impact our findings, as DOX has been reported to contribute to the generation of ROS in cancer cells [[49], [50], [51]]. Although this could conceivably impact our results, significant doxycycline-induced oxidative stress has only been reported at concentrations starting around an order of magnitude higher than the anticipated systemic concentration of doxycycline in mice fed a DOX-diet (625 mg/kg or ppm) [43].

Perhaps the most striking result of this study was that neither the introduction of extra- or intracellular catalase activity to the tumor induced transcriptional changes that would suggest significant changes in the levels of hydrogen peroxide or oxygen (Fig. 4). While it could be that catalase is insufficient in quantity or localization to efficiently exert activity throughout the tumor, it may also be that there is not substantial elevation of hydrogen peroxide levels in these tumors to be depleted or to act as a meaningful source for oxygen generation to reverse hypoxia. Despite confirming long-lived catalase presence and activity at the tumor, we did not attempt to specifically test how and if these tumor-localized catalases impact tumor hydrogen peroxide levels. Therefore, we cannot say with certainty that extra- or intracellular catalase had no impact on hydrogen peroxide flux in the tumor, simply that any changes that did occur did not result in significant transcriptional changes or tumor growth delay.

While therapeutic modulation of hypoxia and oxidative stress cannot be excluded on the basis of our results as viable therapeutic strategies in cancer [11,12,20], our results do contradict a growing body of experimental reports of catalase administration effects [[2], [3], [4], [5], [6], [7], [8], [9], [10]]. We suggest these findings be taken into account when considering any future development of catalase as a cancer therapeutic.

## Author contributions

A.S. and K.D.W. conceived the study and wrote the manuscript; A.S. designed and performed the experiments and analyzed data. H.D.S. aided in editing the manuscript. Y.A., K.M.C., S.C.C., J.A.S., and J.R.P aided in performing the experiments, analyzing data, and editing the manuscript.

## Financial support

A.S., S.C.C., and J.R.P. were supported by the National Science Foundation Graduate Research Fellowship Program.

## Declaration of competing interest

Y.A. and K.D.W. are named as inventors in a patent application filed by the Massachusetts Institute of Technology related to the data presented in this work (US20200405950A1). K.D.W is a co-founder of Ankyra Therapeutics, which has licensed right to the intellectual property mentioned above.

## Data Availability

Data will be made available on request.
