# Supplementary material for: Tumor-localized catalases can fail to alter tumor growth and transcriptional profiles in subcutaneous syngeneic mouse tumor models
Source: Redox Biol. 2023 Jun 5;64:102766. doi: 10.1016/j.redox.2023.102766 (PMC10276214; doi:10.1016/j.redox.2023.102766)
Supplement: Multimedia component 1 [file mmc1.pdf]

Supplementary Information

Supplementary Materials and Methods

Supplementary Figures and Tables

SI Figure 1. Sortagging reaction purification and *in vitro* characterization of catalases
SI Figure 2. Catalase activity in tumor samples
SI Figure 3. *In vivo* efficacy of tumor-localized catalase in MC38 and B16F10 tumors

Supplementary Table 1. Protein amino acid sequences
Supplementary Table 2. List of differentially expressed genes

Supplementary Materials and Methods

Lentivirus production and stable-cell line generation. To generate lentiviral particles, HEK293-FT cells were transfected with pCW57.1-GFP or -CAT,GFP and 2nd generation packaging vectors (Δ8.2 and pMD2, from Addgene) using TransIT-293 Transfection Reagent (Mirus Bio). 4T1 and CT26 cells were transduced overnight with lentivirus in complete media supplemented with 5 ug/mL polybrene. Transduced cells were selected with 4 ug/mL puromycin for 48h, then treated with 2 ug/mL DOX for 24h, after which GFP+ cells were sorted using a MA900-1 cell sorter (Sony).

Sortagging reaction and purification. 50 uM CAT-LPSTG, 1 mM G_4-pTyr_4 peptide, and 5 uM sortase were combined in buffer containing 50 mM Tris-HCl (pH = 7.5), 150 mM NaCl, and 10 mM $CaCl_2$ and left rotating at 4°C overnight (*38*, *39*). The reaction product was then purified by size-exclusion chromatography followed by ion exchange chromatography (HiTrap™Q HP, Cytiva). Endotoxin was removed as described previously. The G_4-pTyr_4 peptide was synthesized by the Koch Institute Swanson Biotechnology Center – Biopolymers and Proteomics core using Tribute peptide synthesizer (Gyros Protein Technologies) and purified by HPLC.

Flow cytometry. Cells engineered to inducibly express CAT and GFP, or GFP only in a DOX-inducible manner were plated, treated with 0-2000 ng/mL DOX for 24h, then analyzed for GFP fluorescence. Cells were analyzed using a BD LSR II flow cytometer. Data were analyzed in FlowJo v10. For comparison between cell lines, GFP intensity was normalized to the average GFP signal at 2000 ng/mL DOX.

Supplementary Figure 1:

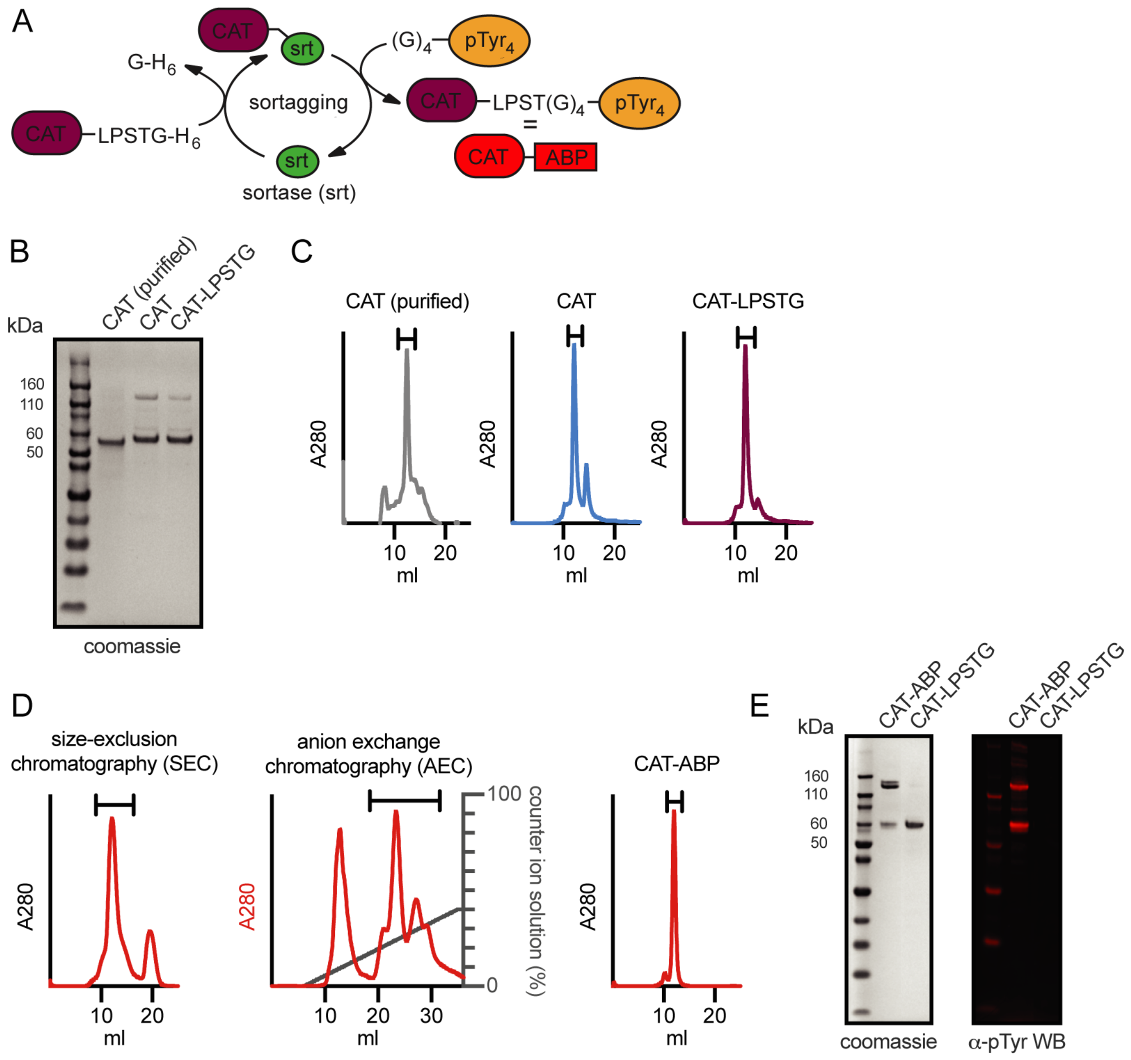

Supplementary Figure 1. Sortagging reaction purification and in vitro characterization of recombinant catalases. (**A**) Schematic of the sortase-mediated transpeptidation ("sortagging") approach used to modify recombinant catalase (CAT) with an alum-binding peptide (ABP), a phosphorylated peptide ($pTyr_4$) that enables long-term binding to the injectable material alum. The sortase enzyme recognizes a C-terminal -LPXTG motif, enabling the site-specific attachment of an N-terminal poly-glycine (G_4) peptide. (**B**) Purified catalases on a non-reducing SDS-PAGE protein gel with Coomassie blue stain (1 ug/protein). (**C**) Size-exclusion chromatograms of 100 μg purified catalases on Superdex 200 Increase 10/300 GL column. Black bars indicate the fraction purified for further analysis. (**D**) Size-exclusion chromatogram of sortagged catalase reaction product (100 μg) on Superdex 200 Increase 10/300 GL column (left) separating CAT-LPSTG and CAT-ABP from sortase and excess peptide based on size (fraction purified = black bar). Anion exchange chromatogram (IEX) of purified fraction from SEC (black bar) (middle) separating CAT-ABP from CAT-LPSTG based on charge (fraction purified

= black bar). CAT is tetrameric and has 4 potential sites to attach ABP therefore multiple peaks indicate different numbers of ABPs attached per tetrameric protein. Size-exclusion chromatogram of SEC and IEX purified sortagging reaction shows a pure product (right). (**E**) Purified CAT-ABP compared to CAT-LPSTG on a non-reducing SDS-PAGE protein gel with Coomassie blue stain (1 μg/protein) (left) and an anti-phosphotyrosine western blot of the same samples (right) showing specific phosphorylation of CAT-ABP.

Supplementary Figure 2:

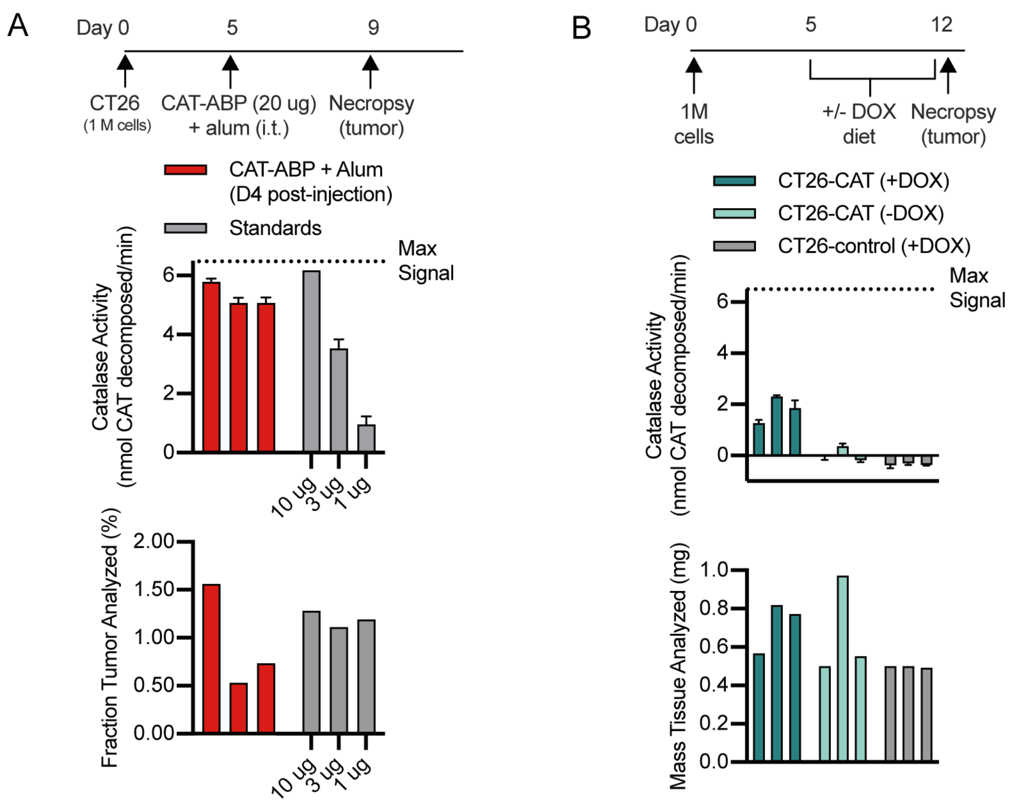

Supplementary Figure 2. Catalase activity in tumor samples. **(A)** Mice were inoculated on day 0 with 1x10^6 CT26 cells and treated i.t. on day 5 with 20 μg CAT-ABP and 100 μg alum or untreated. 4 days after treatment, tumors were excised and homogenized via mechanical disruption in T-PER buffer. After homogenization, standard samples were generated by adding in 1, 3, or 10 μg of CAT-ABP to untreated tumor samples. Unfiltered, uncentrifuged samples were analyzed for catalase activity (*n*=3). Any centrifugation pulls down alum-bound protein and therefore cannot be used to separate out larger tissue fragments. The fraction of total tumor analyzed for catalase activity per sample is shown. **(B)** Mice were inoculated on day 0 with 1x10^6 CT26-CAT or CT26-control cells and started on a DOX diet on day 5. After 7 days, tumors were excised and homogenized and lysed via mechanical disruption in T-PER buffer. Homogenized samples were centrifuged and the supernatant was filtered and analyzed for catalase activity (*n*=3). The mass of tumor tissue analyzed per sample is shown. Note: catalase activity is non-linear as the activity approaches the maximum signal.

Supplementary Figure 3:

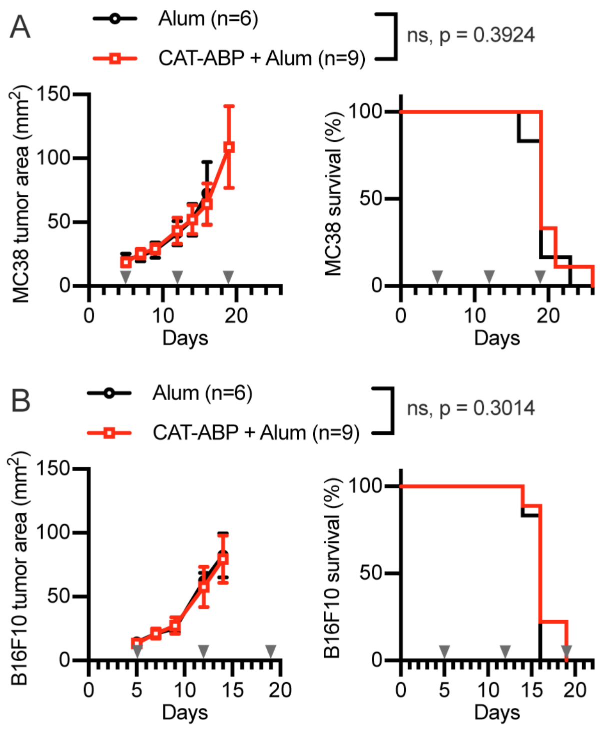

Supplementary Figure 3. *In vivo* efficacy of tumor-localized catalase in MC38 and B16F10 tumor models. Mice were inoculated on day 0 with $1x10^6$ MC38 or B16F10 cells. Mice were treated i.t. on days 5, 12, 19, 26 (triangles above x-axis) with 20 μg CAT-ABP and 100 μg alum or alum alone in 20 μL TBS. Tumor growth and survival for wildtype (WT) **(A)** MC38 and **(B)** B16F10 treated with CAT-ABP and alum or alum only. Statistics shown are for survival. Statistics: survival compared by log-rank Mantel-Cox test. ns, not significant; **P* < 0.05; ***P* < 0.01; ****P* < 0.001.

Supplementary Table 1. Protein amino acid sequences

**Recombinant proteins**	
**Protein**	**Amino acid sequence**
CAT<br>(CAT-H_6)	MADSRDPASDQMQHWKEQRAAQKADVLTTGAGNPVGDKLNVITVGPR<br>GPLLVQDVVFTDEMAHFDRERIPERVVHAKGAGAFGYFEVTHDITKYSK<br>AKVFEHIGKKTPIAVRFSTVAGESGSADTVRDPRGFAVKFYTEDGNWDL<br>VGNNTPIFFIRDPILFPSFIHSQKRNPQTHLKDPDMVWDFWSLRPESLHQ<br>VSFLFSDRGIPDGHRHMNGYGSHTFKLVNANGEAVYCKFHYKTDQGIKN<br>LSVEDAARLSQEDPDYGIRDLFNAIATGKYPSWTFYIQVMTFNQAETFPF<br>NPFDLTKVWPHKDYPLIPVGKLVLNRNPVNYFAEVEQIAFDPSNMPPGIE<br>ASPDKMLQGRLFAYPDTHRHRLGPNYLHIPVNCPYRARVANYQRDGPM<br>CMQDNQGGAPNYYPNSFGAPEQQPSALEHSIQYSGEVRRFNTANDDN<br>VTQVRAFYVNVLNEEQRKRLCENIAGHLKDAQIFIQKKAVKNFTEVHPDY<br>GSHIQALLDKYNAEKPKNAIHTFVQSGSHLAAREKANL–GGGSGGGS–<br>HHHHHH
CAT-LPSTG<br>(CAT- LPSTG-H_6)	MADSRDPASDQMQHWKEQRAAQKADVLTTGAGNPVGDKLNVITVGPR<br>GPLLVQDVVFTDEMAHFDRERIPERVVHAKGAGAFGYFEVTHDITKYSK<br>AKVFEHIGKKTPIAVRFSTVAGESGSADTVRDPRGFAVKFYTEDGNWDL<br>VGNNTPIFFIRDPILFPSFIHSQKRNPQTHLKDPDMVWDFWSLRPESLHQ<br>VSFLFSDRGIPDGHRHMNGYGSHTFKLVNANGEAVYCKFHYKTDQGIKN<br>LSVEDAARLSQEDPDYGIRDLFNAIATGKYPSWTFYIQVMTFNQAETFPF<br>NPFDLTKVWPHKDYPLIPVGKLVLNRNPVNYFAEVEQIAFDPSNMPPGIE<br>ASPDKMLQGRLFAYPDTHRHRLGPNYLHIPVNCPYRARVANYQRDGPM<br>CMQDNQGGAPNYYPNSFGAPEQQPSALEHSIQYSGEVRRFNTANDDN<br>VTQVRAFYVNVLNEEQRKRLCENIAGHLKDAQIFIQKKAVKNFTEVHPDY<br>GSHIQALLDKYNAEKPKNAIHTFVQSGSHLAAREKANL-GGGSGGGS-<br>LPSTG-HHHHHH
ABP<br>(synthetic peptide)	GGGG-$(pTyr)_4$
CAT-ABP<br>(CAT-LPST-ABP)	MADSRDPASDQMQHWKEQRAAQKADVLTTGAGNPVGDKLNVITVGPR<br>GPLLVQDVVFTDEMAHFDRERIPERVVHAKGAGAFGYFEVTHDITKYSK<br>AKVFEHIGKKTPIAVRFSTVAGESGSADTVRDPRGFAVKFYTEDGNWDL<br>VGNNTPIFFIRDPILFPSFIHSQKRNPQTHLKDPDMVWDFWSLRPESLHQ<br>VSFLFSDRGIPDGHRHMNGYGSHTFKLVNANGEAVYCKFHYKTDQGIKN<br>LSVEDAARLSQEDPDYGIRDLFNAIATGKYPSWTFYIQVMTFNQAETFPF<br>NPFDLTKVWPHKDYPLIPVGKLVLNRNPVNYFAEVEQIAFDPSNMPPGIE<br>ASPDKMLQGRLFAYPDTHRHRLGPNYLHIPVNCPYRARVANYQRDGPM<br>CMQDNQGGAPNYYPNSFGAPEQQPSALEHSIQYSGEVRRFNTANDDN<br>VTQVRAFYVNVLNEEQRKRLCENIAGHLKDAQIFIQKKAVKNFTEVHPDY<br>GSHIQALLDKYNAEKPKNAIHTFVQSGSHLAAREKANL-GGGSGGGS-<br>LPST-GGGG-$(pTyr)_4$

Supplementary Table 1. Protein amino acid sequences (continued)

Lentiviral vector protein coding sequences	
**Construct**	**Amino acid sequence**
CAT, EGFP (CAT-T2A-EGFP)	MADSRDPASDQMQHWKEQRAAQKADVLTTGAGNPVGDKLNVITVGPRGPLLVQDVVFTDEMAHFDRERIPERVVHAKGAGAFGYFEVTHDITKYSKAKVFEHIGKKTPIAVRFSTVAGESGSADTVRDPRGFAVKFYTEDGNWDLVGNNTPIFFIRDPILFPSFIHSQKRNPQTHLKDPDMVWDFWSLRPESLHQVSFLFSDRGIPDGHRHMNGYGSHTFKLVNANGEAVYCKFHYKTDQGIKNLSVEDAARLSQEDPDYGIRDLFNAIATGKYPSWTFYIQVMTFNQAETFPFNPFDLTKVWPHKDYPLIPVGKLVLNRNPVNYFAEVEQIAFDPSNMPPGIEASPDKMLQGRLFAYPDTHRHRLGPNYLHIPVNCPYRARVANYQRDGPMCMQDNQGGAPNYYPNSFGAPEQQPSALEHSIQYSGEVRRFNTANDDNVTQVRAFYVNVLNEEQRKRLCENIAGHLKDAQIFIQKKAVKNFTEVHPDYGSHIQALLDKYNAEKPKNAIHTFVQSGSHLAARE-GGGSGGGS-EGRGSLLTCGDVEENPGP-VSKGEELFTGVVPILVELDGDVNGHKFSVSGEGEGDATYGKLTLKFICTTGKLPVPWPTLVTTLTYGVQCFSRYPDHMKQHDFFKSAMPEGYVQERTIFFKDDGNYKTRAEVKFEGDTLVNRIELKGIDFKEDGNILGHKLEYNYNSHNVYIMADKQKNGIKVNFKIRHNIEDGSVQLADHYQQNTPIGDGPVLLPDNHYLSTQSALSKDPNEKRDHMVLLEFVTAAGITLGMDELYK
EGFP	VSKGEELFTGVVPILVELDGDVNGHKFSVSGEGEGDATYGKLTLKFICTTGKLPVPWPTLVTTLTYGVQCFSRYPDHMKQHDFFKSAMPEGYVQERTIFFKDDGNYKTRAEVKFEGDTLVNRIELKGIDFKEDGNILGHKLEYNYNSHNVYIMADKQKNGIKVNFKIRHNIEDGSVQLADHYQQNTPIGDGPVLLPDNHYLSTQSALSKDPNEKRDHMVLLEFVTAAGITLGMDELYK

Supplementary Table 2:

CT26 - EC CAT

Gene	L2FC	p-adj
Tef	-0.836	4.41E-05
Aldoa	-0.37	2.00E-04
Npl	0.832	6.16E-04
Myh4	-0.378	8.17E-03
Ly6c1	-0.544	8.17E-03
Nr1d2	-0.525	1.28E-02
Oasl2	-0.493	1.50E-02
Ifi203	-0.249	1.50E-02
Pgk1	-0.268	1.53E-02
Ubc	-0.198	2.08E-02
Arntl	0.432	2.82E-02
Apol9b	-0.435	2.82E-02
Mir6240	-0.166	2.82E-02
Mest	0.034	3.78E-02
Gpi1	-0.177	4.61E-02
H2-T22	-0.273	4.83E-02
Car9	-0.342	4.92E-02

4T1 - EC CAT

Gene	L2FC	p-adj
no genes with p-adj ≤ 0.05		

4T1 - IC CAT

Gene	L2FC	p-adj
Car4	1.636	8.67E-04
Cd163	1.534	8.67E-04
Ccl9	1.017	8.67E-04
Igkc	1.695	8.67E-04
Dcn	0.731	1.29E-03
Cxcl5	1.321	1.29E-03
Saa3	1.292	1.29E-03
Mir6240	1.622	1.56E-03
Prelp	1.154	3.30E-03
Ly6c2	1.132	3.69E-03
Ccl8	1.183	4.00E-03
Reg3g	1.404	4.00E-03
Serpinb1a	0.978	4.00E-03
Trbc2	1.054	4.68E-03
Gm14419	1.392	5.02E-03
Sell	1.005	5.22E-03
Adamts5	1.051	5.97E-03
Rarres2	0.852	6.14E-03
Cd209d	1.253	6.14E-03
Lpl	1.041	6.20E-03
Ccl6	0.915	6.98E-03
Ckap4	0.494	7.92E-03
Rgs5	0.698	9.53E-03
Gpx7	0.704	9.53E-03
Dyrk3	-0.693	1.12E-02
Pdpn	0.912	1.26E-02
Rnase4	0.46	1.42E-02
Car2	1.005	1.77E-02
Timp1	0.545	1.81E-02
Mcoln2	0.941	1.81E-02
Igf1	0.774	1.81E-02
Cyp1b1	1.015	1.81E-02
Il33	0.979	1.81E-02
H6pd	0.578	1.81E-02
Fabp4	0.84	1.81E-02
Hba-a2	0.822	1.81E-02
Slc29a1	-0.353	1.83E-02
Lims1	0.27	1.92E-02
Cd209a	0.929	1.94E-02
Cxcl12	0.899	1.94E-02
Cd2	0.933	2.05E-02
Slc30a1	0.786	2.19E-02
Col1a2	0.311	2.25E-02
Serpinb2	0.641	2.65E-02
Fut8	-0.352	2.72E-02
Srp9	0.303	2.76E-02
Gm15056	0.794	2.76E-02
Cxcl13	0.559	2.78E-02
Hdc	0.862	2.78E-02
Il13ra2	0.653	2.78E-02
Lilrb4a	0.539	2.78E-02
Chl1	0.844	2.84E-02

4T1 - IC CAT (cont.)

Gene	L2FC	p-adj
Snx29	0.592	2.84E-02
Cpe	-0.258	2.96E-02
Echdc3	-0.697	2.99E-02
Ltbp3	-0.462	3.14E-02
Steap4	0.796	3.28E-02
Fstl1	0.569	3.28E-02
Ms4a2	0.506	3.28E-02
Srp54c	-0.218	3.42E-02
Pgap6	0.737	3.66E-02
Tnnc2	0.741	3.80E-02
Gm49151	0.744	3.80E-02
Serping1	0.503	3.83E-02
Plac8	0.567	3.83E-02
Qars	-0.197	3.83E-02
Cd200r3	0.72	3.89E-02
Nid1	0.421	4.02E-02
Fhl2	-0.305	4.22E-02
Tent5a	0.623	4.22E-02
Flrt2	0.663	4.22E-02
Lix1	-0.506	4.22E-02
Slfn4	0.674	4.24E-02
Col14a1	0.646	4.24E-02
Slc20a1	-0.298	4.24E-02
Myh11	0.635	4.44E-02
Ednrb	0.669	4.44E-02
Emilin2	0.427	4.44E-02
Smc2	0.241	4.44E-02
Ptpru	-0.614	4.44E-02
Dmp1	-0.564	4.44E-02
Hpgd	0.256	4.44E-02
Bmf	-0.656	4.44E-02
Cdc25c	0.625	4.44E-02
Timd4	0.564	4.44E-02
Nrg1	0.543	4.44E-02
Serpina1b	0.651	4.44E-02
Vma21	0.244	4.44E-02
Gm37800	0.656	4.44E-02
Ly6i	0.258	4.52E-02
Gm29336	0.347	4.52E-02
Angptl4	0.495	4.57E-02
Pde4b	0.496	4.57E-02
Fcgrt	0.443	4.67E-02
Serpina3n	0.601	4.67E-02
Hp	0.595	4.67E-02
Ttn	0.609	4.67E-02
Fgr	0.594	4.72E-02
Enpp2	0.574	4.92E-02
Ebf1	0.519	4.92E-02
Plpp3	0.534	4.95E-02
Ankrd66	-0.475	4.95E-02
Lilr4b	0.4	4.98E-02

Supplementary Table 2. Differentially expressed gene lists. List of genes that are significantly (*p*-adj ≤ 0.05) differentially expressed and the associated $\log_2$ fold-change (L2FC) and adjusted *p*-values in **(A)** CT26 + extracellular CAT (CT26 – EC CAT, CAT-ABP + alum vs. alum alone in WT CT26) **(B)** 4T1 + extracellular CAT (4T1 – EC CAT, CAT-ABP + alum vs. alum alone in WT 4T1) and **(C)** 4T1 + intracellular CAT (4T1 – IC CAT, 4T1-CAT + alum vs 4T1-control + alum).
